# Medical Items

**Published:** 1892-05

**Authors:** 


### *MEDICAL ITEMS.*

---

The State Board of Health of Kentucky has been vigorously prosecuting the quacks and traveling doctors in Louisville.

---

The annual meeting of the Association of American Physicians will be held May 24th, 25th and 26th, 1892, in the Medical Museum and Library, Washington, D. C.

---

A Western Exchange is responsible for the following: "The injection hypodermically of perfume is said to be in vogue among the ladies of this and foreign countries. By this means a perfumed perspiration is produced." Why not try it for bromidrosis?

---

Dr. B. Merrill Ricketts, of Cincinnati, seems to be as warm an advocate of circumcision as Dr. Remondino. He circumcises for hygienic reasons, first; also for chancre, chancroids, masturbation, impotence, hip-joint disease, general nervousness, etc. He also makes it his rule to remove the prepuce in every case of gonorrhœa he treats. This in indeed *circoncision à outrance.*

---

The house-surgeon of the Charity hospital, New Orleans, receives a salary of $3,000 per year. He resides in the hospital and is not allowed to do any practice outside. The visiting physicians and surgeons are appointed every six months by the Board of Administrators, upon the recommendation of the house-surgeon. This arrangement, which has been in vogue for a great many years, seems to be occasioning a little friction just at present.

The appropriation for the Surgeon-General's office, at Washington, has been cut down from $10,000 to $5,000. This is much to be regretted, but was done, we suppose, from the intense desire of the present Congress to establish a record for national economy.

---

It is reported that an illustrated journal of preventive medicine will probably make its appearance about July 1, 1892. The plan is to publish it simultaneously in New York, Boston, Philadelphia, Chicago, St. Louis, Atlanta, Kansas City, Denver, San Francisco, Los Angeles and San Diego. The title of the journal will be the *National Popular Review.*

---

Dr. Roberts Bartholow, for so many years the Professor of Materia Medica in the Jefferson Medical College, Philadelphia, and the author of several text-books, is now insane and confined in an asylum. The *Times and Register* says :

"Hard work, no rest, no Sabbath, no vacation; by such means his powerful intellect carried him to the forefront of his profession; but at last outraged Nature reached her limit of endurance, and the breakdown was complete."

---

*Lippincott's Magazine* for May is an unusually attractive number. The "Golden Fleece," by Julian Hawthorne, forms the complete story of this issue and is intensely interesting. The "Intercepted Heiress" is also good. But we enjoyed most the two articles on that quaint but grand old man, Walt Whitman The number is filled with good things.

---

Many thanks to our excellent St. Louis contemporary, the *Weekly Medical Review*, for pleasant allusions to recent changes in the typographical appearance of THE JOURNAL.

They have a curious way of doing things out in Texas. We learn that an interesting and perplexing suit is in progress out there. "A suburban practitioner, called to attend a patient suffering from scarlet fever, advised the landlord to disinfect the house. This was done, and the landlord sued the patient to recover the cost. The latter thereupon sued the doctor for breach of professional secrecy, and it is thought he will win his case."

---

The College of Physicians and Surgeons, of New York, is to have a laboratory of biology constructed upon its grounds at an expense of $100,000. As the money was contributed by the late Charles M. DaCosta, the professor in charge will be known as the DaCosta Professor of Biology. This money comes from Columbia College, and is the first contribution to this medical school, and a forecast of what may be expected in the future. That a great university should take pride in and seek to develop its medical school is the more notable because of its rarity in this country.—*American Lancet.*

---

"FOR SAILE"—A MAD-STONE.—The following letter, directed to "Medical College, Atlanta, Ga.," has been placed in our hands, and is well worthy of publication. We wish it could be printed *fac simile:*

——— ——— KY. JULY 10 91

JENTS—will say that i Have in my care a Rock that weighs 6¼ oz. And is so posed to Be a Mad Stone. And if it should Prove to Be one could it Be disposed of, in that cownty, as i learn that there is Mad dogs in that cownty give me the descriptions of a-Mad Stone, and when this is tested should it prove to Be one if you or any one Else wants to purchase this is for Saile

Yors, —— ———

The following book notice, which we translate from the *Bulletin de Medicine*, Jan. 31, 1892, is striking and interesting:

*Syphilis is Neither Constitutional nor Hereditary;* its Treatment by the Rational Method. By Dr. Joseph Hermann, of Vienna. This monograph is based on an experience of thirty years in the treatment and cure of more than 60,000 patients. "Syphilis is simply a local disease, which never passes into the blood of man, gets well of itself, never leaves any after-effects, and is spread neither by propagation nor heredity. The general health and life of the patient are not injured by it."

---

The Board of Trustees of the Jefferson Medical College, Philadelphia, at their meeting, April 7th, 1892, instituted a Chair of Clinical Gynecology, with a seat in the Faculty and elected to the new Chair, Dr. W. E. Montgomery, who has been for a number of years Professor of Gynecology in the Medico-Chirurgical College. They also established the following clinical professorships, electing Dr. F. X. Dercum, Professor of Nervous Diseases; Dr. E. E. Graham, Professor of Children's Diseases; Dr. H. Augustus Wilson, Professor of Orthopedic Surgery; Dr. H. W. Stelwagon, Professor of Dermatology, and Dr. W. M. L. Coplin, Adjunct Professor of Hygiene.

The College is preparing to erect a new hospital, lecture hall and laboratory building, at a cost of $500,000. The present college buildings are inadequate for the large and increasing attendance of students. With these greatly increased facilities, the Jefferson will do better work than ever before, and continue to be one of the first of American colleges.

